# Features of the response to subchronic low-dose exposure to copper oxide nanoparticles in rats

**DOI:** 10.1038/s41598-023-38976-z

**Published:** 2023-07-23

**Authors:** Marina P. Sutunkova, Yuliya V. Ryabova, Ilzira A. Minigalieva, Tatiana V. Bushueva, Renata R. Sakhautdinova, Ivan A. Bereza, Daria R. Shaikhova, Anna M. Amromina, Aleksei I. Chemezov, Ivan G. Shelomencev, Lev A. Amromin, Irene E. Valamina, Liubov V. Toropova

**Affiliations:** 1 Yekaterinburg Medical Research Center for Prophylaxis and Health Protection in Industrial Workers, 30 Popov Street, Yekaterinburg, Russian Federation 620014; 2 Laboratory of Stochastic Transport of Nanoparticles in Living Systems, Ural Federal University, 51 Lenin Avenue, Yekaterinburg, Russian Federation 620000; 3 Ural State Medical University, 2 Repin Street, Yekaterinburg, Russian Federation 620014; 4 Laboratory of Mathematical Modeling of Physical and Chemical Processes in Multiphase Media, Ural Federal University, 51 Lenin Ave, Yekaterinburg, Russian Federation 620000; 5 Otto-Schott-Institut Für Materialforschung, Friedrich-Schiller-Universität-Jena, 07743 Jena, Germany

**Keywords:** Occupational health, Experimental models of disease

## Abstract

Copper is an essential trace element for human health and, at the same time, a major industrial metal widely used both in its elemental form and in compounds. We conducted a dose-dependent assessment of the response of outbred albino male rats to subchronic low-dose exposure to copper oxide nanoparticles administered intraperitoneally at cumulative doses of 18 and 36 mg/kg during 6 weeks to exposure groups 1 and 2, respectively. We observed disorders at different levels of organization of the body in the exposed animals, from molecular to organismal. The observed decrease in the activity of succinate dehydrogenase in nucleated blood cells gave evidence of impaired bioenergetics processes. In view of the results of the metabolomics analysis, we assume mitochondrial damage and contribution of apoptotic processes to the pathology induced by copper poisoning. We also assume neurodegenerative effects based on the assessed morphological parameters of the nervous system, results of behavioral tests, and a decreased level of expression of genes encoding NMDA receptor subunits in the hippocampus. The hepatotoxic effect noted by a number of metabolomics-based, biochemical, and cytological indicators was manifested by the impaired protein-synthesizing function of the liver and enhanced degenerative processes in its cells. We also observed a nephrotoxic effect of nanosized copper oxide with a predominant lesion of proximal kidney tubules. At the same time, both doses tested demonstrated such positive health effects as a statistically significant decrease in the activity of alkaline phosphatase and the nucleated blood cell DNA fragmentation factor. Judging by the changes observed, the cumulative dose of copper oxide nanoparticles of 18 mg/kg body weight administered intraperitoneally approximates the threshold one for rats. The established markers of health impairments may serve as a starting point in the development of techniques of early diagnosis of copper poisoning.

## Introduction

Copper is an essential trace element^1^. At the same time, there is a wealth of information in the scientific literature about possible mechanisms of its adverse effects on microorganisms^2,3^, warm-blooded animals^4^, and human cells^5^. Copper nanoparticles also demonstrate their toxic characteristics, already proven by numerous in vitro experiments on human cells^6–8^ and animals^9^, and in in vivo studies on warm-blooded animals, such as mice^10^ and rats^11–13^. It has been shown that nanoparticles of copper have a more pronounced toxic effect than its microparticles^14^.

Anreddy (2018) reported that copper oxide nanoparticles (CuO NPs) given to Wistar rats with the doses of 5 and 50 mg/kg body weight per day during 14 days caused a significant dose dependent alterations in antioxidant enzyme activities. The results clearly showed a statistical “decrease in glutathione, catalase, and superoxide dismutase activity, whereas the lipid peroxidation product levels were increased”. The author concluded that oral exposure to CuO NPs caused significant liver toxicity, possibly attributed to oxidative stress^15^. Abdelazeim et al (2020) observed “marked significant elevation in liver enzymes, alteration in oxidant-antioxidant balance and an elevation in the hepatic inflammatory marker” following 2 week administration of a daily oral dose of CuO nanoparticles of 100 mg/kg to rats^16^. Oral exposure to CuO NPs for five consecutive days at doses of 32, 64, and 512 mg/kg body weight per day “…induced changes in hematology parameters, as well as clinical chemistry markers.” Besides, “…histopathological alterations were observed in bone marrow, stomach and liver, mainly consisting of an inflammatory response, ulceration, and degeneration”^17^. The subchronic intraperitoneal exposure of rats to CuO NPs at a dose of 10 mg/kg body weight altered “many functional and biochemical indices for the organism’s status, as well as pathological changes of liver, spleen, kidneys, and brain” and an increase in the DNA fragmentation factor^12^. Using histopathological and immunohistochemical methods, Ghonimi et al (2022) revealed degenerative changes within liver and kidneys up to “severe necrosis of hepatocytes with complete disorganizations of the hepatic rays” following intraperitoneal injection of CuO NPs with doses of 5, 10, and 25 mg/kg body weight/day for 9 days^18^.

Human exposure to copper nanoparticles occurs in many industries, including printing and lithium-ion battery manufacturing^19^. In addition to the obvious purposeful use of these nanoparticles, pyrometallurgical processes are known to generate composite aerosols with nanometer range particles predominating^20^. This has been previously confirmed by our own studies showing the example of the size distribution of particles collected on a membrane filter from the workplace air in the course of blister^21^ and refined^12^ copper smelting.

The current trend towards reduced emissions of industrial pollutants through improvement of technological processes, better efficiency of current and newly installed air filtering systems necessitates testing of health effects of low copper concentrations in toxicological experiments.

## Results

### Integral indicators of toxicity

Changes in integral indicators of the general toxic effect of exposure to copper oxide nanoparticles were dose-dependent (Table 1). The duration of the first entry into the dark chamber in the light-dark box test decreased while the summation threshold index describing the ability of the nervous system to sum up subthreshold impulses increased. No changes in the body weight of the animals or their organs (in g/100 body weight) were registered.

**Table 1:** Changes in integral indices of toxic effects of copper oxide nanoparticles at different dose levels (X̅ ± Sx).

Parameter	Control	Exposure group 1	Exposure group 2
Duration of the first entry into the dark chamber in the light–dark box test, s	103.91 ± 39.89	8.60 ± 2.82 *	7.25 ± 1.91 *
Summation threshold index, s	12.94 ± 0.58	15.52 ± 0.64 *	14.77 ± 0.51*
Initial body weight, g	239.17 ± 4.12	231.67 ± 4.14	230.67 ± 2.91
Final body weight, g	292.92 ± 9.58	270.00 ± 5.16	280.91 ± 6.10
Liver weight, g/100 g bw	2.42 ± 0.45	2.76 ± 0.50	2.53 ± 0.51
Kidney weight, g/100 g bw	0.437 ± 0.077	0.443 ± 0.078	0.432 ± 0.084
Spleen weight, g/100 g bw	0.185 ± 0.033	0.167 ± 0.032	0.200 ± 0.040
Brain weight, g/100 g bw	0.522 ± 0.092	0.547 ± 0.097	0.500 ± 0.097
Heart weight, g/100 g bw	0.223 ± 0.048	0.278 ± 0.055	0.221 ± 0.054
Testis weight, g/100 g bw	0.75 ± 0.16	0.70 ± 0.15	0.69 ± 0.17
Succinate dehydrogenase activity, number of formazan granules per 50 blood lymphocytes	575.78 ± 6.10	507.00 ± 8.12 *	508.75 ± 7.83 *
Leukocytes, 10^9^/L	7.60 ± 0.85	8.58 ± 0.79	9.49 ± 1.17
Erythrocytes, 10^12^/L	8.46 ± 0.72	7.75 ± 0.62	7.37 ± 0.76
Hemoglobin, g/L	172.18 ± 13.30	150.57 ± 4.72	160.25 ± 16.47
Hematocrit, %	20.44 ± 1.61	18.54 ± 1.27	17.19 ± 1.63
Platelets, 10^9^/L	614.18 ± 36.52	659.78 ± 56.62	704.57 ± 38.89
Plateletcrit, %	0.183 ± 0.01	0.2282 ± 0.01 *	0.236 ± 0.02 *
Segmented neutrophils, %	19.09 ± 1.44	18.71 ± 1.13	19.75 ± 1.29
Monocytes, %	5.60 ± 0.16	5.25 ± 0.31	5.71 ± 0.36
Lymphocytes, %	71.45 ± 1.69	68.00 ± 1.13	71.63 ± 1.28
Eosinophils, 10^9^/L	0.156 ± 0.03	0.120 ± 0.06	0.094 ± 0.02
Band neutrophils, 10^9^/L	0.075 ± 0.01	0.058 ± 0.01	0.078 ± 0.02
Segmented neutrophils, 10^9^/L	1.32 ± 0.21	1.21 ± 0.33	1.38 ± 0.34
Monocytes, 10^9^/L	0.401 ± 0.06	0.294 ± 0.07	0.43 ± 0.12
Lymphocytes, 10^9^/L	5.02 ± 0.76	3.94 ± 0.91	5.17 ± 1.36
Platelet distribution width, %	13.85 ± 0.80	14.17 ± 0.48	12.98 ± 0.14 #
Platelets, 10^9^/L	614.18 ± 36.52	659.78 ± 56.62	704.57 ± 38.89
Mean platelet volume, fL	5.97 ± 0.15	6.12 ± 0.15	6.13 ± 0.14
Serum albumin, g/L	43.35 ± 1.64	43.17 ± 1.18	40.63 ± 0.97
Serum globulins, g/L	15.33 ± 4.36	14.31 ± 3.67	17.75 ± 4.28
Atherogenic index of plasma	1.904 ± 0.08	1.775 ± 0.09	1.468 ± 0.07 *#
Serum aspartate aminotransferase activity, U/L	237.58 ± 11.74	239.63 ± 19.33	260.47 ± 14.27
Serum alanine aminotransferase activity, U/L	42.96 ± 2.55	50.37 ± 2.04 *	47.05 ± 3.48
De Ritis ratio	5.08 ± 0.38	4.83 ± 0.48	5.22 ± 0.52
Triglycerides, mmol/L	1.20 ± 0.20	1.25 ± 0.15	0.94 ± 0.11
Cholesterol, mmol/L	1.83 ± 0.18	1.62 ± 0.15	1.740 ± 0.03
High-density lipoproteins, mmol/L	1.31 ± 0.14	1.17 ± 0.11	1.077 ± 0.06
Low-density lipoproteins, mmol/L	0.220 ± 0.04	0.187 ± 0.03	0.264 ± 0.02
Alkaline phosphatase, U/L	199.28 ± 9.45	194.77 ± 19.35	136.47 ± 13.96 *#
Urinary total protein, g/L	232.16 ± 16.08	188.78 ± 51.98	163.05 ± 12.33 *

Significant values are in [bold].

*p* < 0.05 compared with * controls and # exposure group 1.

Under effect of copper oxide nanoparticles, the activity of succinate dehydrogenase (SDH) in blood lymphocytes showed a statistical decrease in both exposure groups whereas plateletcrit demonstrated a dose-related increase and the absolute platelet count tended to grow with the increasing dose of CuO NPs.

We observed changes in biochemical blood serum parameters, including a decrease in the albumin/globulin ratio owing to a decrease in the absolute number of albumins and a slight increase in globulins, a decrease in the activity of alkaline phosphatase (AP), and an increased activity of alanine aminotransferase (ALT).

### Imprint cytology

Dose-dependent changes in the ratio of certain types of cells in imprint smears of liver and kidneys following administration of various doses of copper oxide nanoparticles are shown in Table 2. We observed increased numbers of degenerated hepatocytes in liver, degenerated cells of proximal and distal tubules and eosinophils in kidneys.

**Table 2:** Proportions of different cells in imprint smears of internal organs of rats exposed to copper oxide nanoparticles at different dose levels (X̅ ± Sx).

Cells observed	Controls	Exposure group 1	Exposure group 2
Degenerated hepatocytes in liver smears, %	5.33 ± 0.42	8.33 ± 0.67 *	11.00 ± 0.632 *#
Degenerative cells of proximal tubules in kidney smears, %	6.33 ± 0.49	14.17 ± 0.75 *	14.67 ± 0.49 *
Degenerative cells of distal tubules in kidney smears, %	5.00 ± 0.58	6.17 ± 0.48	6.67 ± 0.33 *
Eosinophils in kidney smears, %	2.67 ± 0.33	4.83 ± 0.31 *	4.33 ± 0.42 *

Significant values are in [bold].

*p* < 0.05 compared with * controls and # exposure group 1.

### Electron microscopy

Samples were taken from three animals of each exposure group (n = 6). We examined 108 micrographs of the liver made by electron microscopy.

**Figure 1:**
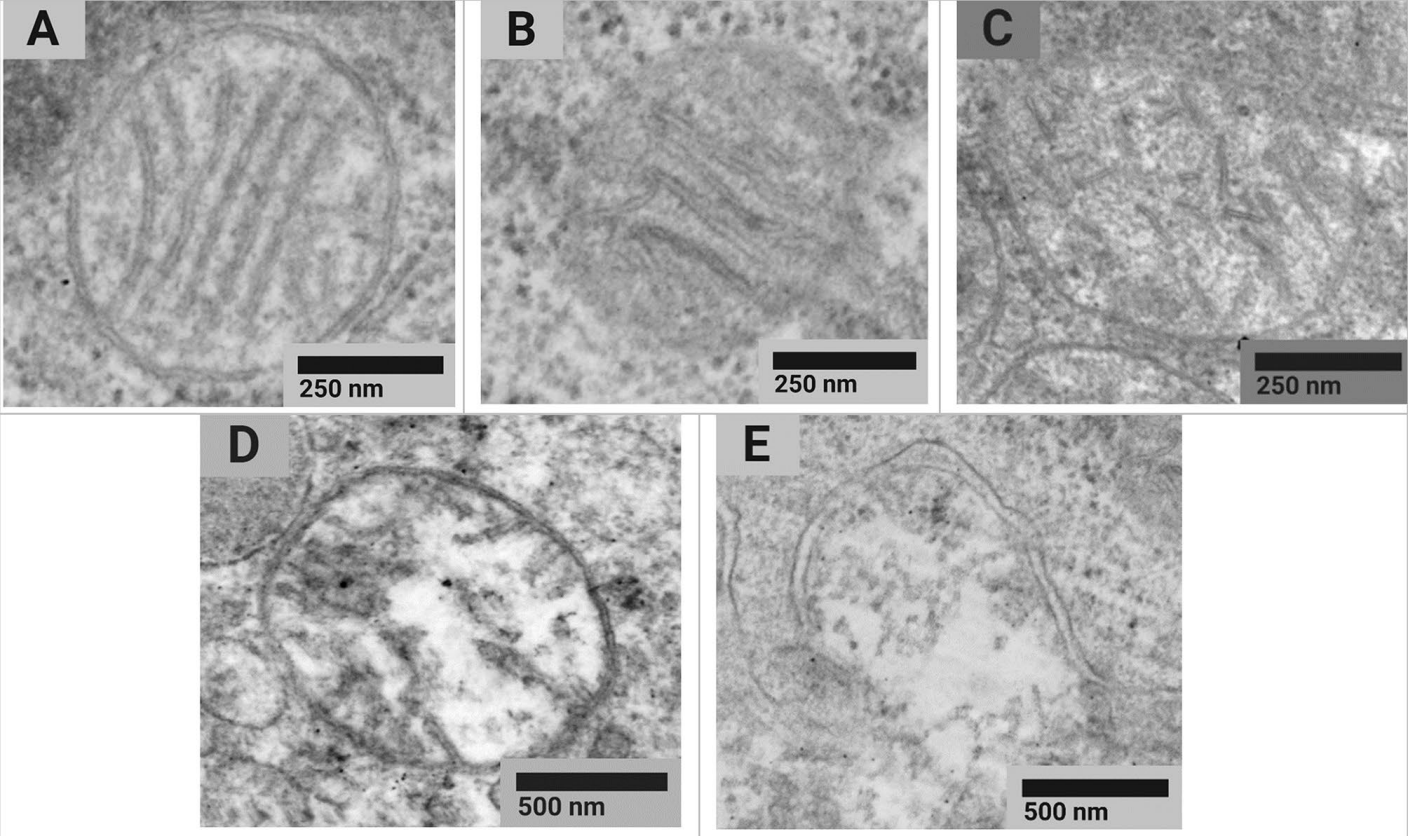
Examples of typical STEM images of normal (**a**), normal vesicular (**b**), vesicular (**c**), swollen vesicular (**d**), and swollen (**e**) mitochondrial morphotypes found in tissues of the control animals and exposure group 2 rats receiving CuO NPs at the cumulative dose of 36 mg/kg bw.

The analysis of the morphotype composition of mitochondria in rat liver cells showed differences between the experimental and control groups (Pearson’s chi-squared test *χ*2 (4; 0.05) = 10.5; *p* = 0.033) (Fig. 1).

However, the Pearson’s chi-squared goodness of fit test (χ2) compared distribution of mean group values regardless of their intragroup variability. Pairwise comparison of the percentage of each of the morphotypes in the exposure and control groups showed no significant differences in the Mann-Whitney U test (Table 3).

**Table 3:** Results of studying the ultrastructure of mitochondria in rat liver cells (morphotyping of mitochondria according to Sun, 2007).

Group	Morphotype percentage of the total number of mitochondria (Mean ± SE), %				
	Normal	Normal vesicular	Vesicular	Vesicular swollen	Swollen
Control	87.0 ± 0.4	7.8 ± 1.1	2.0 ± 1.0	2.6 ± 0.9	0.6 ± 0.3
Exposure group 2	83.2 ± 1.7	8.0 ± 0.3	5.6 ± 1.7	1.2 ± 0.1	2.0 ± 0.4

Thus, we noted a tendency to manifestation of cytotoxicity at the level of mitochondrial ultrastructure, namely: a slight decrease in the proportion of normal and an increase in damaged mitochondria across the entire spectrum with the exception of vesicular swollen ones.

### Metabolomics activity screening

**Figure 2:**
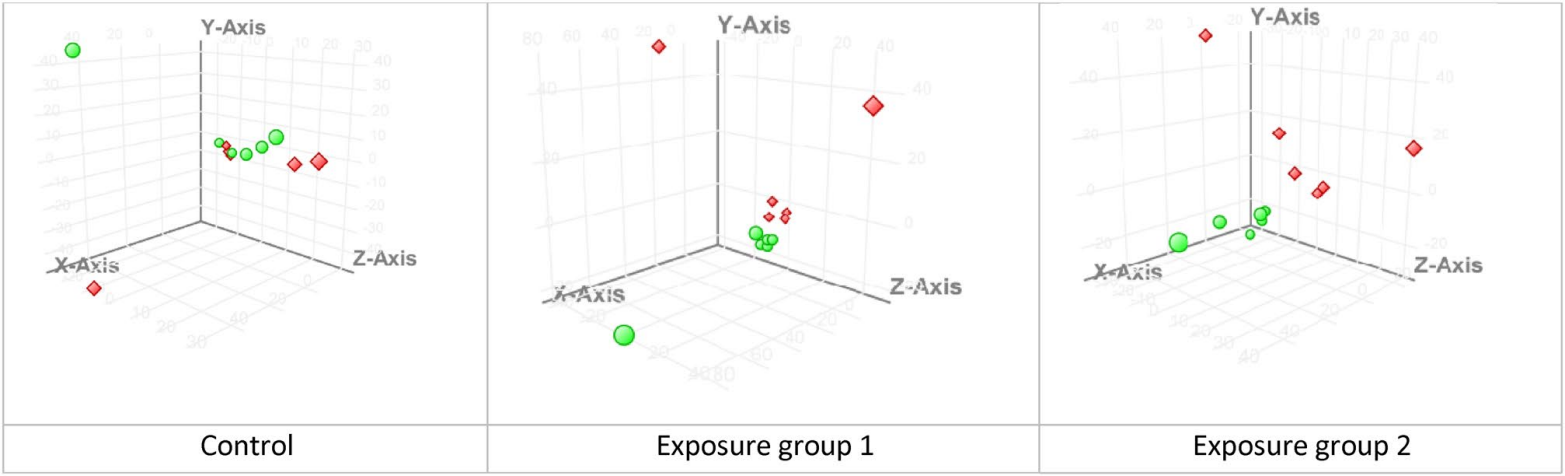
Results of HPLC–MS data analysis of blood sample spectra using the principal component analysis (● before and ♦ after the exposure).

Results of the principal component analysis showed the absence of sample clustering in the control group. The observed divergence of samples in the exposure groups was dose dependent (Fig. 2). Moreover, the Student's *t*-test demonstrated that the number of metabolites that significantly changed their content was significantly higher in the second (higher dose) exposure group.

**Figure 3:**
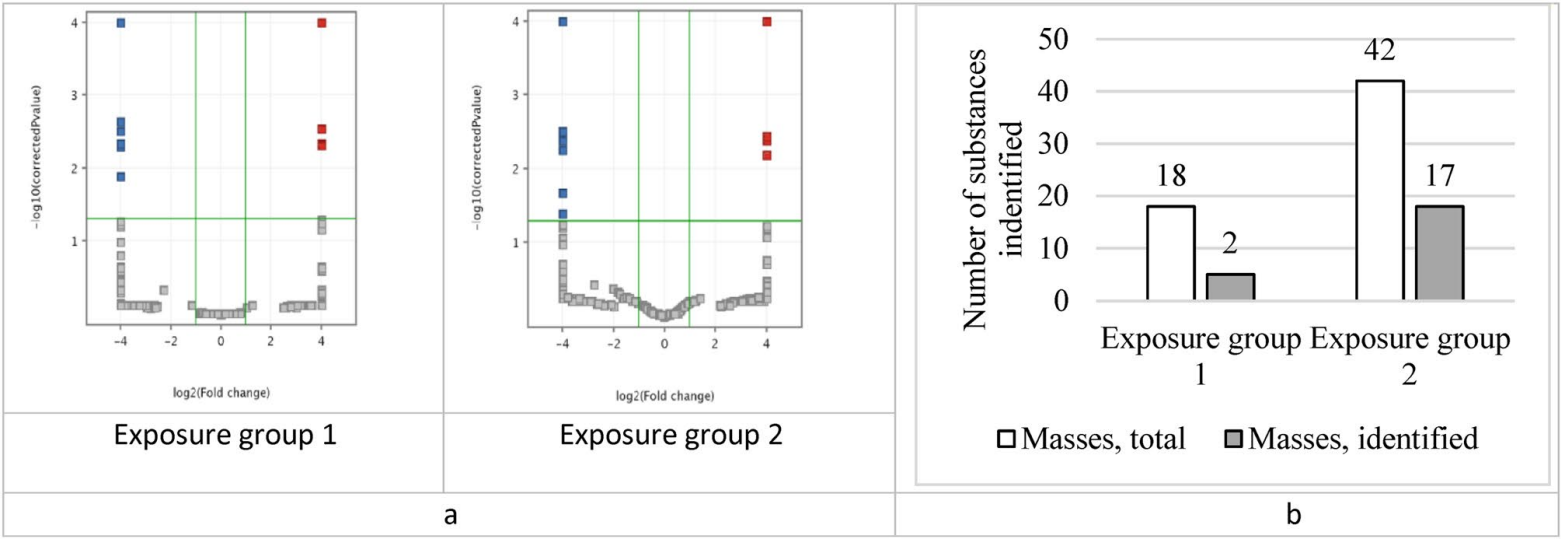
(**a**) A volcano plot of the *t*-test results: the substances marked with red and blue squares showed a statistical change in concentration after the exposure; (**b**) the number of substances with the changed concentration in exposure groups obtained from the *t-*test adjusted for multiple comparisons.

The statistical analysis of data using the principal component method and the multiple comparison-adjusted *t*-test in the Mass Profiler Professional software revealed statistically significant changes in both exposure groups (Fig. 3).

Only a part of the selected substances had sufficient intensity of the analytical signal to obtain informative fragment spectra, so the annotation was not possible for all metabolites in the groups. Table 4 shows the list of annotated metabolites.

**Table 4:** Results of the metabolomics-based blood test in the rats after subchronic exposure to copper oxide nanoparticles.

#	Exposure group	m/z	Quasi-molecular ion	Annotated substance	Retention time	*p*	Fold change	Database substance ID
1	1	623.3177	[M+Na]+	LPI(18:0)	16.94	0.0067	5.28	HMDB0240261
2		332.3324	[M+H]+	Hexadecylbenzylamine	18.01	0.0205	4.84	CID15416275
3	2	568.2653	[M+Na]+	LPS(20:4)	14.57	0.0460	2.62	LMGP03050007
4		502.2943	[M+H]+	LPE(20:4)	14.91	0.0308	2.13	HMDB0011488
5		502.2943	[M+H]+	LPE(20:4)	15.25	0.0186	4.14	HMDB0011488
6		583.3242	[M–H_2_O+H]+	LPI(18:0)	16.97	0.0027	12.37	HMDB0061704
7		643.2845	[M+Na]+	LPI(20:4)	14.00	0.0283	7.74	LMGP06050006
8		595.2847	[M+Na]+	LPI(16:0)	14.87	0.0283	8.70	HMDB0061695
9		295.2644	[M+H]+	Methyl linoleate	23.61	–	–	HMDB0034381
10		271.2633	[M+H]+	Methyl hexadecanoic acid	24.86	0.0040	5.07	HMDB0061859
11		429.3729	[M–H_2_O+H]+	alpha-Tocopherolquinone	27.08	0.0007	2.66	HMDB0034408
12		331.2859	[M+H]+	MG(16:0/0:0/0:0)	19.89	0.0009	− 2.11	HMDB0011564
13		359.3170	[M+H]+	MG(0:0/18:0/0:0)	22.31	0.0009	− 2.35	LMGL01010003
14		359.3159	[M+H]+	MG(18:0/0:0/0:0)	22.73	0.0009	− 2.31	LMGL01010003
15		239.2380	[M–H_2_O+H]+	Palmitic acid	19.89	0.0009	− 2.03	HMDB0000220
16		468.3094	[M+H]+	LysoPC(14:0/0:0)	14.05	0.0409	− 2.12	HMDB0010379
17		398.2301	[M+H]+	LPC(9:0)	17.83	0.0457	− 2.70	LMGP01050068
18		552.4043	[M+H]+	LysoPC(20:0/0:0)	21.78	0.0429	− 3.66	HMDB0010390
19		643.2845	[M+Na]+	LPI(20:4/0:0)	14.34	0.0283	7.74	HMDB0061690

Abbreviations: *HMDB* Human Metabolome Database, *LM* LipidMaps, *CID*, PubChem Compound Identification.

The changes were likely to be dose-dependent. The level of lysophosphatidylcholines (LPC) decreased whereas those of LPE, lysophosphatidylinositols (LPI), and lysophosphatidylserine (LPS) increased. The level of monoglycerides and palmitic acid decreased and those of methyl hexadecanoic acid and methyl linoleate increased.

### Estimation of the genomic DNA fragmentation factor

Both doses tested statistically decreased the DNA fragmentation factor in nucleated blood cells to 0.398725 + 0.000357 in exposure group 1 and to 0.399505 + 0.000215 in exposure group 2 against 0.425771 + 2.24E-05 in the controls, *p* < 0.05.

### Gene expression analysis

Table 5 shows data on genetic alterations in the exposed animals. The highest of the doses tested caused a decrease in the expression of GRIN1 gene in the hippocampus of the experimental rats while that of GRIN2A and GRIN2B genes demonstrated a dose-dependent decline.

**Table 5:** Indicators of genetic alterations under effect of copper oxide nanoparticles at different dose levels (X̅ ± Sx).

Indicator		Control	Exposure group 1	Exposure group 2
Level of expression of genes encoding NMDA receptor subunit proteins in the hippocampus	GRIN1, GluN1 subunit proteins	1.1 ± 0.078	0.46 ± 0,077	0.101 ± 0.06*
	GRIN2A, GluN2A subunit proteins	1.17 ± 0.361	0.693 ± 0,08	0.497 ± 0.13 *
	GRIN2B, GluN2B subunit proteins	1.18 ± 0.795	0.642 ± 0,1	0.343 ± 0.05 *
Genomic DNA fragmentation factor for nucleated blood cells		0.425771 ± 0.00002	0.399505 ± 0.00022*	0.398725 ± 0.00036*

Significant values are in [bold].

*p < 0.05 compared with controls.

## Discussion

We have already studied subchronic toxicity of CuO nanoparticles following repeated intraperitoneal injections in rats at the doses of 10 mg/kg b w.^12^ and 2.5 mg/kg bw.^13^ per day. We observed hematological and biochemical changes in blood, histological changes in liver and kidneys, accumulation of copper in kidneys, and neurodegenerative disorders in the study with a high dose of copper. Subsequent experiments with a lower dose showed a decrease in specific manifestations of copper poisoning. The doses chosen for this study are interesting in terms of the search for more subtle changes at the metabolome and genome levels induced by copper oxide nanoparticles.

### Suppression of bioenergetics processes

Mitochondria are known targets for almost all types of damaging agents, including toxins^22^ and oxidative stress^23^. A change in the potential of the mitochondrial membrane under effect of copper nanoparticles has been reported^6^. Mitochondrial dysfunction is often difficult to measure and prove^24^; yet, it can be assumed by alterations in certain markers.

Succinate dehydrogenase (SDH) is a mitochondrial enzyme attached to its inner membrane. It is involved in the tricarboxylic acid cycle and the electron transport chain in mitochondria by linking these two processes^25^. SDH dysfunction is of great importance in medical practice as it causes a wide specter of disorders: from neurogenerative^26^ to carcinogenic^27,28^, by disrupting mitochondrial functioning and reducing the bioenergetic potential of the cell.

The exposure to CuO nanoparticles caused a decrease in SDH in exposure groups 1 and 2 by 11.95 % and 11.64 %, respectively. In our previous experiments, we also registered a decrease in the activity of this enzyme by 20.72 % at a single dose of 2.5 mg/kg^13^ and by 12.23 % at a single dose of 10 mg/kg^12^.

Based on the assumption that functional changes in mitochondria related to their disruption can be detected by electron microscopy, we searched for alterations at the subcellular level. An ultrastructural study of the liver showed a decline in the proportion of normal type A mitochondria. At the same time, such an effect was not observed for the sum of types A and B, both of which could be attributed to normally functioning mitochondria.

### Early signs of toxicity

The functions of mitochondria are diverse; they include oxidative phosphorylation for the production of cellular adenosine triphosphate (ATP), involvement in ion homeostasis, some metabolic pathways, programmed cell death, production and consumption of reactive oxygen species^29^. Changes in mitochondria representing cellular “energy stations” are important for the development of pathological conditions of organs and systems and of the whole organism later on.

The metabolomics-based blood testing of the exposed animals showed a relatively slight increase in the level of lysophosphatidylserine (LPS (20:4)), a metabolite that triggers inflammatory processes and acts as a signaling substance in the process of the uptake of apoptotic cells by macrophages^30^. This finding may indicate both the inflammatory process and enhanced apoptosis in the exposed animals.

Apparently, if the inflammatory process does exist, then it is extremely insignificant, since the general blood test results showed no statistical differences between the cases and controls in terms of inflammatory processes, except for the platelet count (Table 1). Platelets serve as an important link between the coagulation and immune systems ^31^. A statistically significant increase in the plateletcrit and a trend towards an increase in the number of platelets may indicate inflammation related to production of reactiveoxygen species (ROS) induced by the exposure to CuO NPs^32,33^. ROS, in their turn, are mediators of activation of the immune system^34^. A slight increase in the level of globulins in exposure group 2 was likely to occur owing to an increase in fractions of alpha-1-globulin and beta globulin for the same reason.

A 1.8-fold increase in the eosinophil count indirectly indicates the ability of nanosized copper oxide to stimulate immunocompetent cells, as demonstrated by Tulinska et al.^35^.

In view of the above, we assume contribution of apoptotic changes to the developing pathology. Apart from the increase in the level of lysophosphatidylserine, apoptosis was evidenced by a decrease in the proportion of normal mitochondria and an increase in all types of damaged mitochondria, except for vesicular swollen ones (Table 3). Data on the topology of the inner membrane correlated with the physiological state of mitochondria in the course of cell death, i.e. with the loss of the potential of the mitochondrial membrane, which could be accompanied by a release of cytochrome. We established that the vesicular configuration was associated with the release of proteins and cytochrome^36,37^. Further transition of mitochondria to the swollen configuration occurs only after the loss of potential of the mitochondrial membrane, a long time after the release of cytochrome^38^.

### Effects on select organs and systems

Mitochondria are one of the key components of apoptosis in mammalian cells^39^. A relationship between abnormal apoptosis and certain diseases, including neurodegenerative conditions (Alzheimer’s, Huntington’s, and Parkinson’s diseases distinguished by excessive apoptosis of neurons) has been noted by now^40^.

### Effects on the nervous system

Ionotropic glutamate NMDA receptors are responsible for fast synaptic transmission and play an important role in regulating duration of the excitatory potential^41,42^. In combination with a decrease in the brain weight, we may assume neurodegenerative conditions in the exposed animals (Table 5).

This supposition was confirmed by the results of behavioral testing: a rising summation threshold index indicated the predominance of inhibitory processes in the nervous system^43^. An increased anxiety level related to the nanoparticle exposure was judged by a decrease in the duration of the first entry into the dark chamber in the light–dark box test^18,19^.

### Hepatotoxic effects

We have also found signs of liver damage, presumably mediated by excessive apoptosis. The metabolomics screening showed a decrease in the level of lysophosphatidylcholines, previously noted following the exposure to ions of some heavy metals, including copper^44,45^. Since these substances are synthesized in liver, a decrease in their blood levels can be attributed to an impaired synthetic function of this organ in relation to LPC. This impairment was also proven by a lower albumin to globulin ratio due to a lower albumin count attributed either to suppression of its synthesis or its increased losses, exemplified by a renal disorder accompanied by proteinuria ^46^. Yet, we found a decrease in the level of urinary total protein suggesting some suppression of albumin synthesis in liver under effect of CuO NP exposure (at a dose of2 mg/kg). We have described similar suppression of the protein synthesizing function following the exposure to CuO NPs at a single dose of 2.5 mg/kg elsewhere^13^.

We noted a significant decrease in the activity of alkaline phosphatase (AP) in the second exposure group at a single dose of 2 mg/kg. We observed a similar effect of exposure to CuO NPs at a single dose of 2.5 mg/kg^13^, but not at 10 mg/kg^12^; besides, it is a classic symptom of the Wilson’s disease, a congenital defect of copper metabolism leading to its accumulation^47^. A lower AP activity is associated with zinc and magnesium deficiency since both metals are part of this enzyme^48^. Yet, an impaired AP activity was observed at a subchronic exposure to ZnO NPs^13^, enabling us to assume a key role of the zinc to magnesium ratio in the AP activity. Further studies are necessary to clarify the causes and mechanisms of reducing alkaline phosphatase activity.

A change in the secretory function of the liver was also evidenced by a lower (compared to controls) number of triglyceride cleavage products, i.e. monoglycerides MG(16:0) and MG(18:0), palmitic acid, as well as a higher level of esters of hexadecanoic and linoleic acids. Triglycerides and fatty acid esters are cleaved by hepatic lipase synthesized in this organ and released in the bloodstream; as a result, a decrease in the secretion of this enzyme is expected, which is also supported by an increase in the level of lysophosphatidylethanolamine LPE(20:4), the direct effect of which on liver cells inhibited the expression of genes responsible for the synthesis of lipases^49^. At the same time, indicators of the blood lipid profile of the exposed animals (triglycerides, cholesterol, low and high-density lipoproteins) were not significantly different from those in the controls.

**Figure 4:**
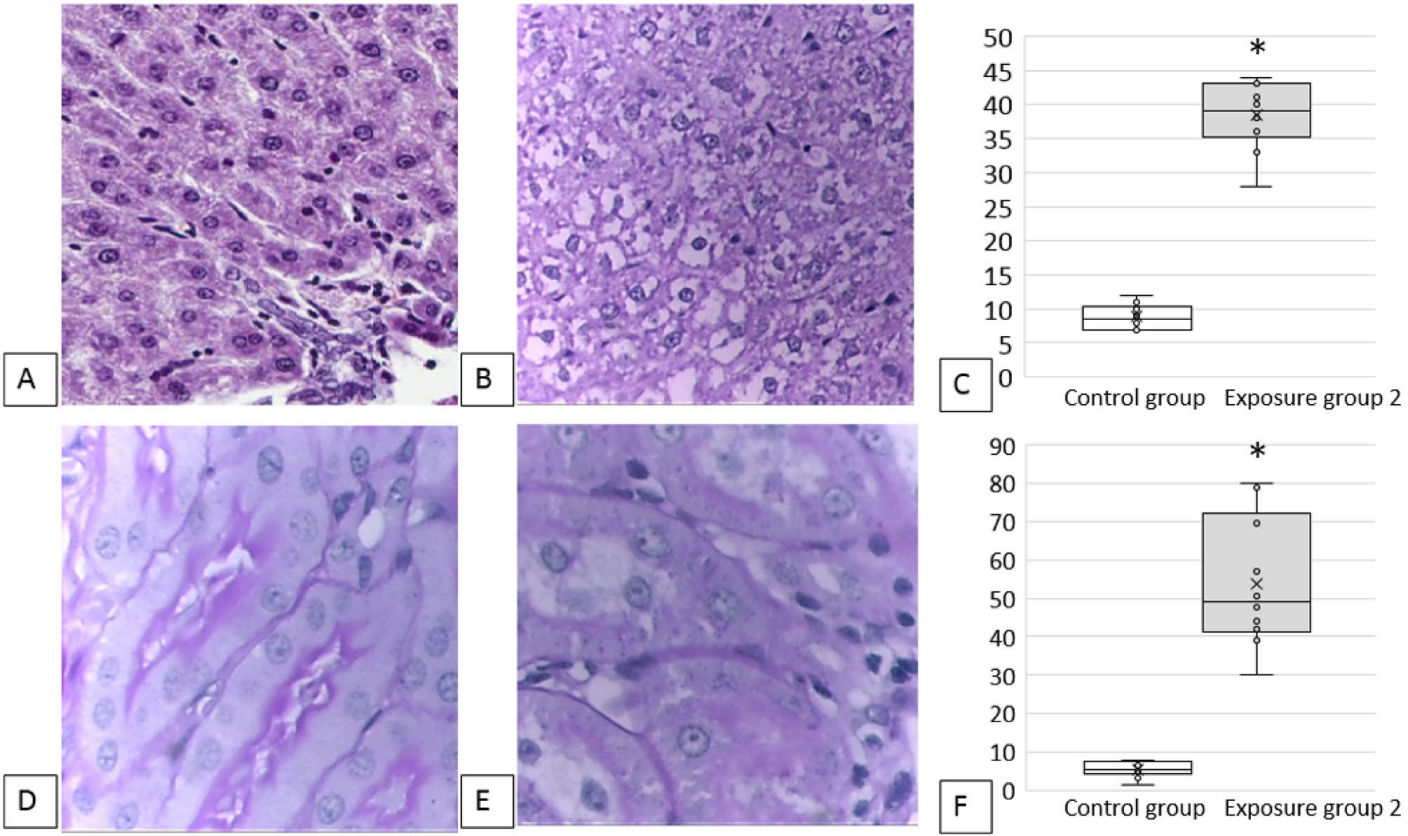
Results of a histological examination of the experimental animals: (**A**) liver, control group: normal structure of liver, 100 × magnification; (**B**) liver, exposure group 2: pronounced dystrophic and necrobiotic changes in hepatocytes, 100 × magnification; (**C**) the increased number of prokaryotic hepatocytes, %, * *p* < 0.05 compared with controls; (**D**) kidneys, control group: the epithelium of convoluted tubules of kidneys with a clear PAS-positive brush border on the apical edge, the cytoplasm is homogeneous, the nuclei are well visualized, the lumens of the tubules are not dilated, 100 × magnification; (**E**): kidneys, exposure group 2: foci of destruction of the PAS-positive brush border of the tubular epithelium, dystrophic changes in the cytoplasm, tubular luminal dilation, 100 × magnification; (**F**) loss of the brush border of the renal tubular epithelium, %, * *p* < 0.05 compared with controls.

The activity of alanine aminotransferase (ALT) has changed, which may also indicate liver damage in the exposed groups. Changes at the molecular level and in biochemical blood parameters were supported by cytological ones: after the exposure to copper oxide nanoparticles, the number of degenerated hepatocytes in imprint smears of liver increased (Table 2) similar to the proportion of prokaryotic liver cells (Fig. 4C). In addition, we observed marked dystrophic and necrobiotic changes in hepatocytes (Fig. 4B) in the exposed animals compared with the controls (Fig. 4A).

### Renal effects

The nephrotoxic effect of CuO NPs was analyzed by the cytology image of kidney imprint smears (Table 2). It is known that proximal tubules are damaged to a greater extent than the distal ones^50^ owing to a different functional load on these parts. According to our findings presented in Table 2 and those described elsewhere^51^, this statement is also relevant for the effects of nanoparticles.

A statistically significant increase in the eosinophil count was observed in the imprint smears of kidneys in both exposure groups. Our findings are consistent with those published by Cho et al.^52^, indicating the ability of copper oxide nanoparticles to induce inflammation involving eosinophils. It is worth noting the potential role of eosinophils in additional damage to kidney cells: activated eosinophils may induce oxidative stress causing cell death^53^.

Histologically, we observed such pronounced morphological changes as dystrophy of renal cells, tubular luminal dilation, and foci of destruction of the PAS-positive brush border of the tubular epithelium (Fig. 4E) compared with the controls (Fig. 4D). The morphometric analysis showed the loss of the brush border of the renal tubular epithelium (Fig. 4F).

### Impact on the genomic DNA fragmentation factor

Previously, the effect of subchronic toxicity of nanosized copper oxide at the genetic level was demonstrated, manifested by a slight but statistically significant increase in the genomic DNA fragmentation factor by 7.58% in liver cells, by 24.66% in spleen cells ^12^, and by 2.12% in nucleated blood cells of rats^13^ compared to the controls. The ability of nanosized copper oxide to damage DNA have been also confirmed by other studies. It has been demonstrated on the HUVEC cell culture, for instance, that copper ions released from nanosized copper oxide can induce oxidative stress and activation of p38 MAPK signaling pathway and cause subsequent DNA damage and cell death^33^. Their anticancer activity is also closely related to ROS generation^54^.

Against the background of an extensive evidence base, it seems paradoxical that, in contrast to the increase in the genomic DNA fragmentation factor almost always observed by us in experiments with nanoparticles of different metal oxides, including those of CuO^12,13^, in the present study we observe more of a protective effect of the latter. This effect can be determined by the fact that modern toxicology has accumulated evidence of non-linear patterns of the dose–response relationship in addition to classical dependencies. According to Erofeeva (2014), under effect of various pollutants, including heavy metals, the majority (85.7%) of dose–response relationships were precisely nonlinear. At the same time, hormesis was noted in 5.5% of cases whereas the rest 94.5% were paradoxical effects, of which multiphase dependencies similar to vibrational ones prevailed^55^.

### Conclusions

Our findings indicate that subchronic exposure to copper oxide nanoparticles at doses of 1 and 2 mg/kg body weight instilled thrice a week during 6 weeks has a toxic effect at the systemic and organismic levels, although not always dose-dependent.

We revealed dose-dependent changes in the rats when assessing the intensity of cellular energy processes, behavioral reaction in rats expressed in the predominance of inhibitory process and a higher anxiety level, and plateletcrit.

The hepatotoxic effect noted by a number of metabolomics-based, biochemical, and cytological indicators was manifested by the impaired protein-synthesizing function of the liver and enhanced degenerative processes in its cells. We also observed a nephrotoxic effect of nanosized copper oxide with a predominant lesion of proximal kidney tubules.

A dose of copper oxide nanoparticles equaling 1 mg/kg body weight administered intraperitoneally 18 times during 6 weeks can be considered as that approximating the threshold one.

The established markers of health disorders may serve as a starting point in the development of techniques of early diagnosis of copper poisoning.

## Methods

### Preparation of suspension and physicochemical characteristics of copper oxide nanoparticles

**Figure 5:**
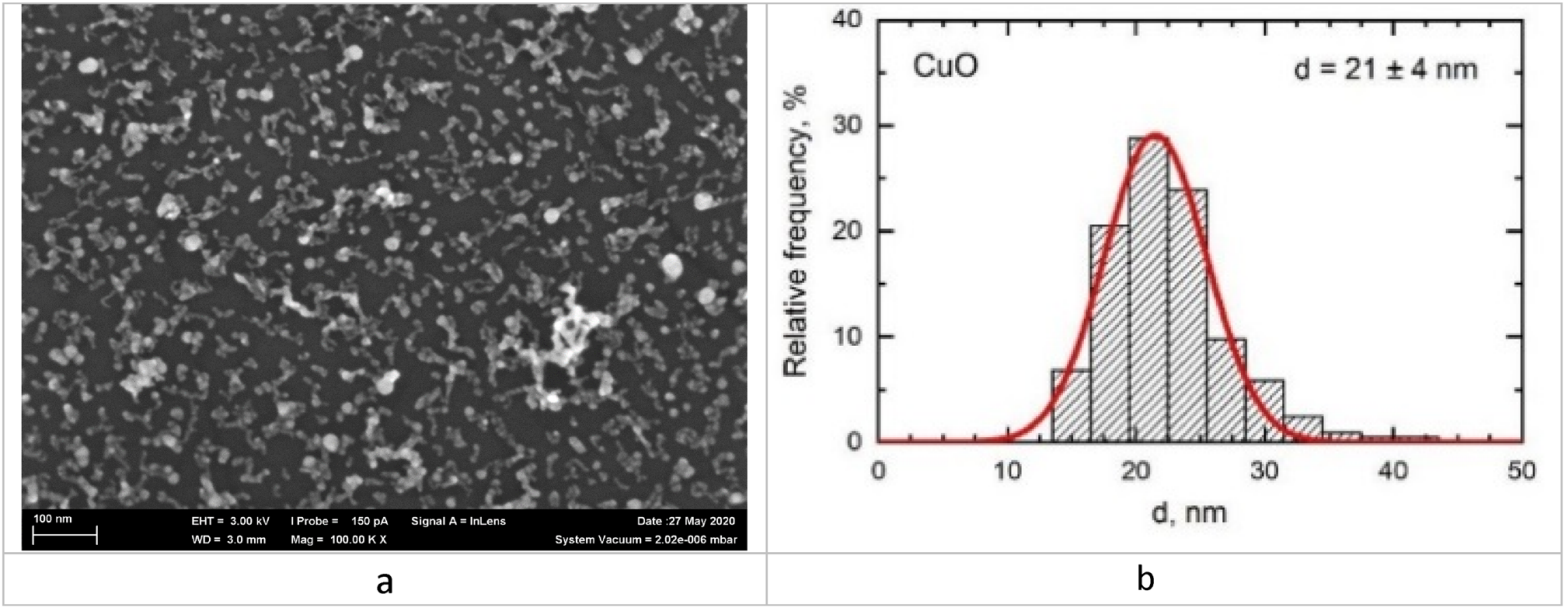
(**a**) Suspended copper oxide nanoparticles (electron microscopy at 100,000 × magnification); (**b**) the particle diameter distribution function showing that the mean diameter of CuO NPs was 21 ± 4 nm.

The suspension of copper oxide nanoparticles was prepared at the Ural Federal University. We used pulsed laser ablation of thin metal sheet targets of 99.99% pure copper in sterile deionized water. The mean diameter of the suspended copper oxide nanoparticles was 21 ± 4 nm (Fig. 5), as shown by scanning electron microscopy (SEM). The suspension stability was judged by the zeta potential measured using the Zetasizer Nano ZS size analyzer (Malvern Panalytical, UK) and was found to be high (up to 42 mV, deionized water), enabling us to increase the particle concentration to 0.25 mg/mL by partial water evaporation at 50 °C without changing the size and chemical identity of nanoparticles.

### Experimental animals

The study was conducted on outbred albino male rats, 12 animals in each exposure group. The initial body weight of the animals was 200–270 g with the range within ± 20% of the mean weight. The age of the rodents at the beginning of the experiment was 12–14 weeks out of concerns for a low survival rate of young adults in this subchronic toxicity study of presumably very hazardous nanoparticles.

The rats were kept in a specially equipped vivarium room in compliance with the International Guiding Principles for Biomedical Research Involving Animals developed by CIOMS and ICLAS (2012). They were euthanized by rapid decapitation with isoflurane anesthesia used additionally for painless beheading. Study approval was provided by the Local Ethics Committee of Yekaterinburg Medical Research Center for Prophylaxis and Health Protection in Industrial Workers, protocol No. 2 of April 20, 2021.

### Justification of the doses and route of administration

Subchronic toxicity was modeled by repeated intraperitoneal injections thrice a week during six weeks (18 instillations in total). Deionized water was injected to control animals; 2 mL of stable suspensions of CuO nanoparticles at the concentrations of 0.125 mg/mL and 0.25 mg/mL were injected to exposure group 1 and 2 animals, making up single exposure doses of 1 and 2 mg/kg and the cumulative doses of 18 and 36 mg/kg following 18 instillations, respectively.

The choice of the exposure dose was determined by the results of a literature search showing that intraperitoneal instillation of copper oxide nanoparticles at the doses of 5 to 25 mg/kg bw. per day for nine days caused liver and kidney changes and even tissue necrosis^18^. Oral administration of 5 mg/kg bw. of CuO NPs per day for 14 days to Wistar rats induced significant alterations in antioxidant enzyme activities and liver toxicity^15^. Besides, we relied on our own previous experience showing that 10 mg/kg bw. of these NPs significantly altered various indices of the organism^12^ while 2.5 mg/kg bw. had no effect^13^.

The intraperitoneal route of administration were chosen for greater accuracy in individual dosing.

### Exposure assessment

On the fifth week of exposure, we registered the summation threshold index^56^ and conducted a light–dark box test^43,56,57^. After exposure cessation, we visually examined macroscopic changes in internal organs and weighed them; we also analyzed biochemical parameters of blood serum. The bioenergetic state of the rats was determined by the activity of succinate dehydrogenase (SDH) established cytochemically using nitro blue tetrazolium chloride and expressed as the number of formazan granules per 50 cells using optical microscopy with immersion oil^58^. Blood parameters were determined using a Mythic 18 hematology analyzer.

### Imprint cytology

For imprint cytology, imprint smears were made from cross-sections of liver and kidneys, dried at the room temperature, and then stained by Leishman stain. The cellular composition and signs of cell damage were analyzed using a Carl Zeiss Primo Star light binocular microscope with a USCMOS video camera visualization system at 100 × and 1000 × magnifications. During microscopy, we counted 100 cells from each smear of lymph nodes and 300 cells from smears of other organs.

### Histological studies

Histological specimens were prepared by immersing kidneys and liver in formalin, then cutting them into 2–3 mm thick slices treated with alcohols of increasing concentration, and embedding in paraffin. Then, 3–4 μm sections were cut from the embedded blocks and stained with hematoxylin and eosin; in addition, the method of periodic acid for kidneys –Schiff stain was also used. The study of histological preparations, their microphotography and morphometry were carried out using the Avtandilov ocular measuring grid and a computer software for pattern recognition^59–61^ using an Olympus CX-41 microscope with an Olympus Soft Imaging Solution GMBH, Model LC20 camera and LCmicro software. At least 30 measurements of each indicator were taken on preparations of four rodents from each exposure and control groups.

### Electron microscopy

Cell ultrastructure was assessed using scanning transmission electron microscopy (STEM). The degree of damage to mitochondria was determined according to the classification by Mei G. Sun based on morphological characteristics, such as matrix space and the number of cristae^36^. In calculations, mitochondria of types A (normal) and B (normal vesicular) were counted as normal whereas those of types C (vesicular), D (vesicular swollen), and E (swollen) were considered abnormal.

### Metabolomic screening

Metabolomic screening was performed using high performance liquid chromatography-mass spectrometry (HPLC–MS) with a time-of-flight mass spectrometer. Raw data was processed with Agilent MassHunter software package for mass spectra processing and feature extraction. Data processing was performed in Mass Profiler Professional; the statistical analysis included PCA and paired t-test. For each exposure group, we obtained a set of m/z values, which intensity altered statistically during the experiment. Those masses were annotated by exact mass and fragment spectra analysis using available databases (HMDB, MoNA, METLIN, MassBank EU) and in silico fragmentation tools (MetFrag, CFM‑ID, MS-FINDER).

### Degree of genomic DNA fragmentation

The degree of genomic DNA fragmentation was assessed in the Central Research Laboratory of the Ural State Medical University, Yekaterinburg, Russian Federation, by analyzing amplified fragment length polymorphism on nuclear cells of circulating blood as described elsewhere^62^.

### Gene expression analysis

To analyze gene expression, a part of the rat hippocampus was fixed in IntactRNA to stabilize RNA in bioassays with the following phenol/chloroform RNA extraction. The quantitative RNA analysis was performed using a Qubit™ 4 fluorometer and the Qubit™ RNA BR Assay Kit. RNA samples were treated with RNase-free DNase I followed by a reverse transcription reaction with a MMLV-RT kit using a SimpliAmp™ Thermal Cycler. Determination of expression of GRIN1, GRIN2A, and GRIN2B genes encoding GluN1, GluN2A, and GluN2B proteins, respectively, was carried out by real-time polymerase chain reaction (RT-PCR) with primers, probes^63^, a ready-made mixture qPCRmix-HS by Evrogen, using a Quant StudioTM 3 RT-PCR system. The level of gene expression was established by the level of mRNA of the studied gene relative to that of the reference “housekeeping” GAPDH gene using the delta delta CT (ΔΔCt) method.

### Statistical data processing

The statistical significance of data on behavioral reactions, the genomic DNA fragmentation factor, and biochemical parameters was assessed using the Student’s t-test (*p* ≤ 0.05). In addition, we compared *p* values estimated by the Student’s *t*-test and the Mann–Whitney test, and their general coincidence proved the appropriateness of applying the *t*-test.

The statistical SEM data processing was performed using the Statistica software by StatSoft. The significance of differences between groups was determined using the Student’s *t*-test, Mann–Whitney U-test, and a one- and two-way ANOVA.

For statistical processing of data related to gene expression, a nonparametric Kruskal–Wallis test was used for pairwise comparison of several groups in the Statistica 12 software.

The difference between mean values was considered statistically significant if the probability of its random occurrence was less than or equal to 0.05 (*p* ≤ 0.05).

### Ethical approval

All methods were carried out in accordance with relevant guidelines and regulations. The manuscript reporting adheres to the ARRIVE guidelines for the reporting of animal experiments. The study was approved by the Ethics Committee of the Yekaterinburg Medical Research Center for Prophylaxis and Health Protection in Industrial Workers (protocol No. 2 of April 20, 2021).

## Data Availability

The data presented in this study are available on request from the corresponding author.
